# RainDrop: Rapid activation matrix computation for droplet-based single-cell RNA-seq reads

**DOI:** 10.1186/s12859-020-03593-4

**Published:** 2020-07-01

**Authors:** Stefan Niebler, André Müller, Thomas Hankeln, Bertil Schmidt

**Affiliations:** 1 Department of Computer Science, Johannes Gutenberg University, Mainz, 55099 Germany; 2 Molecular Genetics and Genome Analysis, Institute of Organismal and Molecular Evolution, Johannes Gutenberg University, Mainz, 55099 Germany

**Keywords:** Single-cell sequencing, RNA, Locality sensitive hashing, Big data

## Abstract

**Background:**

Obtaining data from single-cell transcriptomic sequencing allows for the investigation of cell-specific gene expression patterns, which could not be addressed a few years ago. With the advancement of droplet-based protocols the number of studied cells continues to increase rapidly. This establishes the need for software tools for efficient processing of the produced large-scale datasets. We address this need by presenting RainDrop for fast gene-cell count matrix computation from single-cell RNA-seq data produced by 10x Genomics Chromium technology.

**Results:**

RainDrop can process single-cell transcriptomic datasets consisting of 784 million reads sequenced from around 8.000 cells in less than 40 minutes on a standard workstation. It significantly outperforms the established Cell Ranger pipeline and the recently introduced Alevin tool in terms of runtime by a maximal (average) speedup of 30.4 (22.6) and 3.5 (2.4), respectively, while keeping high agreements of the generated results.

**Conclusions:**

RainDrop is a software tool for highly efficient processing of large-scale droplet-based single-cell RNA-seq datasets on standard workstations written in C++. It is available at https://gitlab.rlp.net/stnieble/raindrop.

## Background

Droplet-based single-cell RNA-seq (dscRNA-seq) protocols have gained increasing attention due to their ability to profile the transcriptome of thousands of cells in a single assay. Information about gene expression in terms of cDNA counts within certain cells is a crucial processing step for further analysis such as clustering [1, 2] or imputation [3]. The gained knowledge can be key to many biological research areas; e.g. the identification of genetic differences between cancerous and non-cancerous cells [4] or finding connections between different cell types [5, 6]. Thus, the creation of gene-cell-count matrices from dscRNA-seq data is of high technical importance. For each cell this matrix shows the estimated count of genes within that cell based on the number of reads mapping to corresponding transcript sequences. Since the number of studied cells continues to increase this can be an enormously time-consuming task. Thus, efficient processing of the generated data is critical.

However, existing software tools often suffer from scalability issues due to the massive amounts of reads. For example, calculating a gene-cell expression matrix (also called feature-barcode matrix) with the popular Cell Ranger pipeline [7] can take several hours even for a medium-sized input read dataset. This pipeline is based on alignments using STAR [8] which are expensive to calculate. Srivastava et al. [9] recently used a different approach in their tool Alevin by relying on quasi-mappings using a suffix array based structure [10]. They showed that this approach produces similar results while only requiring a fraction of the time compared to Cell Ranger. However, for larger datasets Alevin still needs a significant amount of time. Since the number of studied cells continues to increase, corresponding runtime and memory requirements will become even more severe, especially for ultra-large datasets such as the recently published 1.3 million-cell dataset [11].

We present RainDrop, a fast tool for the computation of gene-cell count matrices from dscRNA-seq data produced by 10x Genomics Chromium v2 protocols. Our approach avoids compute-intensive alignments by employing fast *k*-mer lookups to a subsampled precomputed hash table based on minhashing. This allows RainDrop to process droplet-based sequence datasets in a fraction of the time needed by state-of-the-art tools while maintaining high agreements on expressed genes per cell with established methods like Cell Ranger and Alevin. RainDrop also shows high Spearman correlation compared to bulk RNA-seq data. Moreover, index creation in RainDrop is significantly faster and more memory efficient in comparison to Alevin. Similar subsampling strategies have already been successfully applied in metagenomics; e.g. [12, 13]. We are the first to apply such a method in the area of mapping single-cell sequencing reads, whereby RainDrop is based on the scheme used by MetaCache [13]. The classification method has been adjusted to work with transcriptomic data in order to account for the higher amount of *k*-mer ambiguity caused by alternative splicing. We have additionally inserted the mapping of transcripts to their corresponding genes into the database. Further important enhancements include the processing of Chromium v2 protocols, whitelisting of valid cell identifiers, and deduplication.

## Implementation

RainDrop accepts a set of reads sequenced under a 10x Genomics Chromium v2 protocol as input and generates a gene-cell count matrix showing gene expression for individual cells within the sample. In this protocol, cells are distinguished by tagging with a so-called cellular barcode (CB). In general, the capture rate of mRNA in dscRNA-seq experiments is relatively low [14]. This can be compensated for by performing many rounds of PCR amplification, which may in turn skew the distribution of molecules per cell. As a countermeasure, each molecule is also tagged by a unique molecular identifier (UMI). Similar to the CB this can be used to identify unique molecules and to correct PCR amplification bias. Input reads are in paired-end format with the first mate containing a fixed length CB and UMI string. These two codes can be used to assign a read to a unique cell and to a molecule within that cell. The second mate contains the actual read data which can be mapped to a gene. While CBs and UMIs are drawn from a predetermined set of possible codes only a fraction of those will ultimately appear in a dataset. The true set of valid codes is therefore unknown during processing and has to be extracted from the data in order to ensure correct mapping of reads.

RainDrop requires a transcript database for querying together with a corresponding mapping of transcripts to genes in form of a two-level taxonomy. This database (stored as a hash table) is created in a preprocessing step from a given FASTA file containing all reference transcripts of a transcriptome. Furthermore, the transcript-to-gene mapping needs to be generated by extracting this information from corresponding GTF files.

**Fig. 1:**
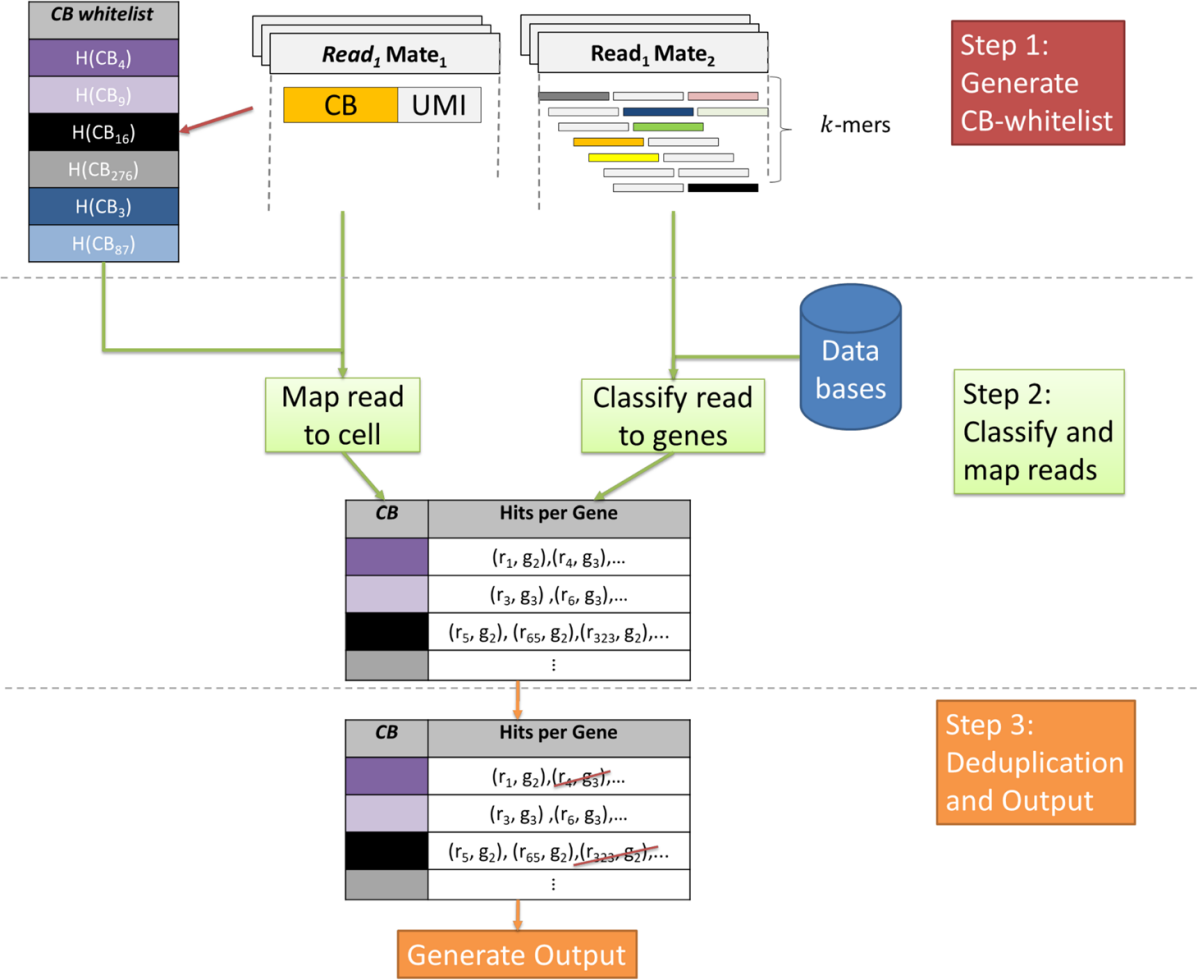
General workflow of RainDrop

The overall workflow of RainDrop is illustrated in Fig. 1. It consists of three processing stages as well as an output stage. In the first stage a whitelist of valid CBs is created by extracting all CBs which occur above a certain threshold. In the second stage each read is mapped to a cell using its CB before mapping it to genes using the transcript-to-gene mapping information. Furthermore, reads whose CB cannot be matched within an edit distance of one to a whitelisted CB are discarded. In the third stage all mappings within a cell are deduplicated using the directional method [15] and the output is generated. A total of three output files is written to disk. One file contains the gene-cell count matrix in sparse COO format while the other two files contain a mapping from genes/cells to coordinates of the matrix.

In the following we will present the individual stages in more detail.

### Database creation

**Fig. 2:**
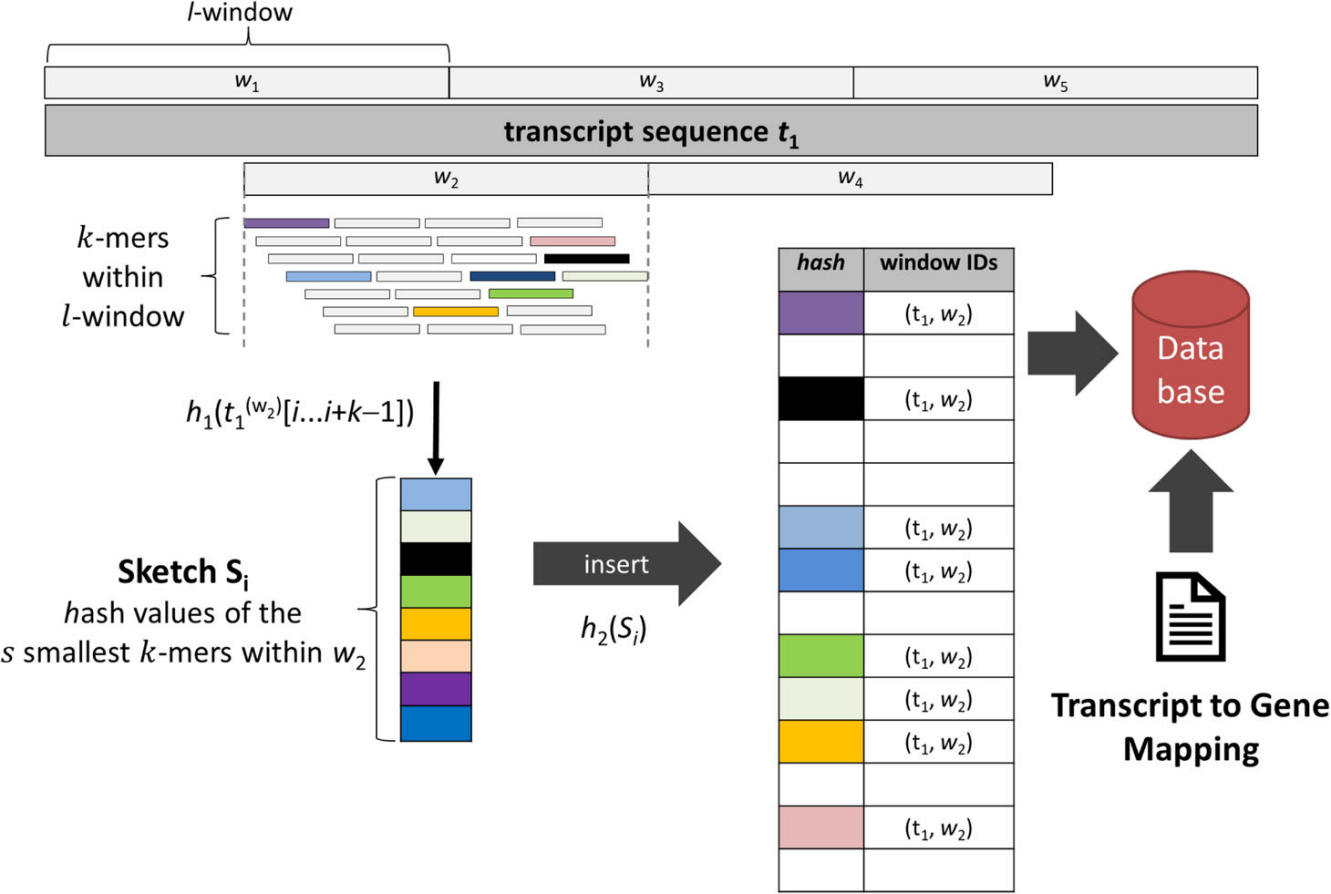
Pre-processing stage of RainDrop

Reads are mapped to a set of transcripts by adapting an approach based on minhashing of *k*-mers, a strategy previously applied to metagenomic read classification [13] As shown in Fig. 2, a hash table (key-value store) containing a subset of *k*-mers (keys) from each transcript is created. Each transcript is distributed into *m* windows *W*_*m*_ with a certain stride *t* and size *w*. The union of all windows covers the whole transcript. In general we recommend choosing a stride and size such that neighboring windows are not disjoint either. As default the stride is chosen to be *t*=*w*−*k*+1. This way each possible *k*-mer is considered, however, windows only share a *k*-mer if it occurs on different positions within the same transcript. To reduce memory consumption, each window is represented by a so-called sketch consisting of a small number of features which are based on a subset of all *k*-mers within that window. A sketch is created by first applying a suitable hash function *h*_1_ to all *k*-mers within a window and then selecting the *s* smallest *k*-mer hash values as features. The sketch of each window is inserted into a hash map using a secondary hash-function *h*_2_. As a feature may occur in multiple transcripts or windows this results in a multi-value hash table. Features that occur in too many transcripts or windows may be discarded to avoid overflowing the hash table value store and to improve performance during the read mapping phase.

The resulting hash table is written to disk together with auxiliary data structures to form a database that allows for the mapping of features to transcripts and transcripts to genes. Transcript-to-gene mapping is enabled by a two-level taxonomy generated from a GTF annotation file.

### Whitelist generation

A whitelist of CBs is generated at the beginning of each sample analysis in order to estimate the amount of cells in the sample and to determine which CBs represent these cells. A CB consists of several predefined nucleotides and serves as a unique identifier for the cell of origin. As CBs are sequenced along with the read data, they might be affected by sequencing errors, resulting in altered barcodes. As a consequence reads may be assigned to wrong cells which in turn would lead to a distorted gene cell matching result. As the true CBs are unknown we need to rely on heuristics to identify them. We assume that truly expressed CBs will be present multiple times and we can therefore estimate the list of all valid CBs, called the whitelist, by discarding rare CBs. Barcodes are sorted by how often they occur in descending order and a prefix sum over the number of occurrences is calculated. All CBs whose prefix sum is below a predefined fraction of all reads are considered valid and will be added to the whitelist of this sample. The remaining bar codes are considered faulty. In the mapping stage, we will try to assign them to one of the whitelisted CBs by searching for a match within an edit distance of one.

### Mapping stage

**Fig. 3:**
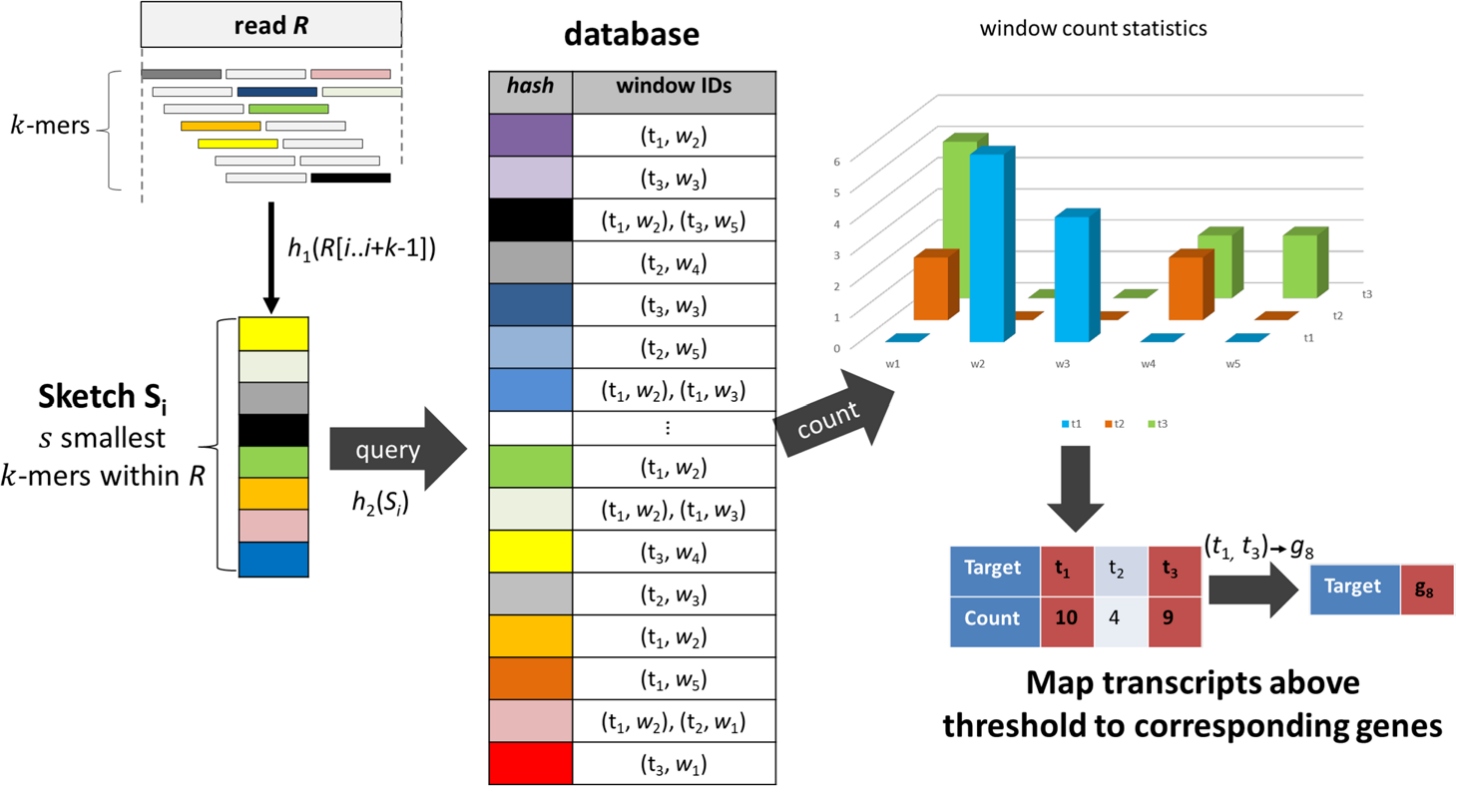
Mapping stage of RainDrop

During mapping, both mates of the sequencing data are read from disk and CBs are matched with the whitelist to identify cells of origin. If a CB cannot be found in the whitelist, RainDrop looks for a match within an edit distance of one. If such a match is found the corresponding read is assigned to the corresponding cell. If no match is found the CB is considered erroneous and the whole read is discarded. After selecting a cell the read is mapped to a set of transcripts. A given read is split into *m* windows *W*_*i*_, $i < m, i \in \mathbb {N}$ and for each window *k*-mers are extracted as a sketch using hash function *h*_1_ (see Fig. 3). These extracted features are looked up in the database to locate transcript windows that contain similar features. We assume that a read consists of consecutive base pairs within a transcript. As a result hits in consecutive windows within a predefined range of size *r* are accumulated. Hits that are spread out too far will not be aggregated in order to avoid spurious hits. The resulting maximum count of shared features within any range of consecutive windows is the mapping score of the given read to a certain transcript. If this score exceeds a given threshold we consider the transcript to be a mapping candidate.

Candidate transcripts are mapped to corresponding genes. Due to spurious hits and alternative splicing, a read may have several candidate transcripts. Multiple transcripts identified as candidates create conflicts. There are two possible cases that can occur:

1. All identified transcripts map to the same gene. In this case the conflict can be resolved by simply mapping the read to this gene. The reported mapping score for this gene is set to the maximum mapping score for each candidate transcript.
2. A read maps to multiple transcripts that correspond to different genes. While it might be possible to assign the read to all identified genes with certain weights, we decided to discard these ambiguous reads instead. As a result we only output counts for reads that map to a single gene.

Figure 3 summarizes the read mapping stage.

### Deduplication

In the final stage we deduplicate the identified genes in each cell to correct for PCR amplification bias which may lead to certain genes being over-expressed. We deduplicate all valid matches per unique cell using the directional approach proposed by UMI-tools [15]. With this method we can determine reads originating from the same molecule. As we do not want to count a molecule within a cell multiple times we can merge those genes into a single one such that the distribution of genes per cells is less prone to PCR amplification bias. As UMIs may also be erroneous, we match all UMIs within an edit distance of one. In this case we only consider them to be the same UMI if we find one UMI in this matching that has a higher occurrence by a given factor. This means that similar UMIs are kept separate if there is no clear indication that one is only created through sequencing errors as a faulty UMI should be expressed significantly fewer times than the correct UMI on which it is based.

### Output

RainDrop outputs a gene-cell-count matrix in sparse coordinate (COO) format. A coordinate pair (*i*,*j*) indicates that the gene corresponding to column *j* is expressed in the cell corresponding to row *i*. RainDrop provides two additional output files mapping cell names and gene names to rows and columns, respectively. The order of genes is given by the order of which they appear in the provided taxonomy. The order in which the cells are written is random and influenced by the execution order of the threads.

### Parallelization

Participating CPU threads read batches consisting of the first mate of the reads and create local maps counting how often a CB appears within their batch. Individual results are then merged into a global CB-count map and the thread reads the next batch, if available. Once all batches are processed a single thread sorts the resulting map and calculates the threshold to determine the whitelist.

In the mapping stage, data is again read from file and each thread maps its batch independently of other threads before merging it into a global buffer. This buffer is distributed among all threads such that each thread deduplicates a batch of cells identified by their CBs. The final result is written to disk. As each step depends on the results of the previous step synchronization barriers are necessary in-between individual stages (whitelisting, mapping, deduplication). Reading/writing and processing phases of the data are interleaved to minimize idle CPU time during disk IO.

## Results

We compared the performance and accuracy of RainDrop to Cell Ranger v.3.1.0 [7] and Alevin v.0.13.1 [9]. All methods were evaluated on four different datasets, downloaded from the 10x Genomics website created under the 10x Chromium v2 protocol sequenced by an Illumina HiSeq4000 (Illumina HiSeq2500) with a read length of 98. Two of the datasets originate from mouse brain cells (neurons_900, neuron_9k) and two from human (t_4k, pbmc8k). Reference transcriptomes were generated from the mm10-3.0.0 genome for mouse and the GRCh38-3.0.0 genome for human using RSEM (rsem-prepare-reference) [16]. RSEM creates the transcriptome by combining the information from a given genome and genome transcript annotation file. Additionally RSEM is able to create index structures for common aligners such as Bowtie2.

RainDrop uses a sketch size *s*=16 and *k*=16 as default. The window size for a read of length *R* is determined as *w*=(*R*+*k*−1)/2 (56 for the given datasets). Additionally, we limit each feature to a maximum of 1000 locations within the reference to remove unnecessary overhead from processing highly ambiguous features. The mapping threshold parameter is set to 28. We run Alevin during indexing with the --keepDuplicates flag. Apart from that Alevin was executed using default parameters and the --dumpCsvCounts flag to write the output while for Cell Ranger we chose parameters according to the proposed values supplied with the datasets and additionally provided the flags --localmem=128 to limit the memory used by Cell Ranger to 128GB and --no-secondary, which excludes all secondary analysis which is usually performed by Cell Ranger. The limitation of memory should not affect the runtime of Cell Ranger as it is more than the measured maximum memory required by Cell Ranger to evaluate the data sets.

All tests have been performed on a system with an Intel Xeon E5-2683v4 CPU with 256GB of DDR4 RAM running Ubuntu 16.4. Input files are read from SSD. All methods were executed using 16 threads.

### Runtime and scalability

Table 1 shows the runtimes of each method for the four tested datasets. Note that preprocessing for index or database creation is excluded from these runtimes (as this only needs to be done once per reference transcriptome). RainDrop is fastest in all cases with a maximum (average) speedup of 30.4 (22.6) compared to Cell Ranger and 3.5 (2.4) compared to Alevin.

**Table 1:** Runtime for different datasets and 16 threads. Datasets are ordered by genome and size

Dataset	neurons_900	neuron_9k	t_4k	pbmc8k
# Reads	52.8M	383.4M	335.2M	784.1M
Filesize	18.3GB	145GB	117GB	273GB
RainDrop	1m59s	19m24s	14m50s	36m10s
Alevin	6m55	32m15s	33m31s	84m36s
Cell Ranger	60m23s	350m26s	291m21s	804m17s

We further investigated the scalability of RainDrop with respect to the number of CPU threads for the neurons_900 and neurons_9k datasets (see Table 2). Starting from 2 Threads the scaling of RainDrop is close to linear. Compared to the single thread variant RainDrop scales less well as our parallelization requires locks and synchronization barriers which generate an additional overhead.

**Table 2:** Runtimes (Speedup) of RainDrop for different numbers of CPU threads on the *neurons*_900 and *neurons*_9*k* dataset

Method	RainDrop	
Dataset	neurons_900	neurons_9k
1 Threads	18m42s (1)	189m15s (1)
2 Threads	10m58s (1.67)	128m12s (1.48)
4 Threads	5m42s (3.21)	59m34s (3.18)
8 Threads	3m16s (5.62)	31m55s (5.94)
16 Threads	1m59s (9.25)	19m24s (9.76)

Table 3 compares database/index creation times and corresponding memory consumption of RainDrop and Alevin for the two utilized transcriptomes. RainDrop’s indexing structure (hash table) can be build three time faster than the suffix array used by Alevin while consuming only half the amount of memory.

**Table 3:** Runtime and memory consumption for database/index creation

Method	RainDrop		Alevin	
Dataset	mm10-3.0.0	GRCh38-3.0.0	mm10-3.0.0	GRCh38-3.0.0
Time	0m54s	1m03s	2m55s	3m35s
Size	1.3GB	1.5GB	2.6GB	3.2GB

### Mapping quality

We evaluated mapping quality in terms of

1. Agreement between different tools (RainDrop, Cell Ranger, Alevin) and
2. Spearman correlation of identified genes compared to a bulk dataset processed by RSEM and Bowtie2.

*Cell-agreement* between two tools is calculated as the average count of genes found present within a cell by both tools divided by the amount of genes found present in a cell by at least one tool. For this test we first determined all cells identified by both methods and calculated the corresponding confusion matrices. We additionally filtered these matrices such that genes on which the tools disagree on the occurrence in a cell, only those remain that express a hit count greater than 1.0. Table 4 shows the resulting confusion matrices for the comparison between RainDrop to Alevin and between RainDrop and Cell Ranger. Agreement values were derived from these filtered matrices. Compared to Cell Ranger (Alevin) an agreement of at least 98.3*%* (95.2*%*) is achieved for all datasets. It can be seen that RainDrop and Cell Ranger identify almost the same genes in a cell while Alevin identifies slightly more.

**Table 4:** Cell-agreement matrix (*C*) of each method compared to RainDrop for different datasets. Datasets are ordered by genome and size. Columns of the matrices show the average value of genes present (left) or absent (right) in cells calculated by RainDrop. Rows show the average gene count present (top) or absent (bottom) in cells calculated by CellRanger (Alevin)

Method	$C_{neurons\_900}$	$C_{neuron\_9k}$	$C_{t\_4k}$	*C*_*pbmc*8*k*_
Alevin	$\left [\begin {array}{ll}2197&56 \\ 12&28788\end {array}\right ]$	$\left [\begin {array}{ll}2151&42 \\ 15&28846\end {array}\right ]$	$\left [\begin {array}{ll}965&39 \\ 6&32528\end {array}\right ]$	$\left [\begin {array}{ll}1321&60 \\ 6&32151\end {array}\right ]$
CellRanger	$\left [\begin {array}{ll}2205&31 \\ 14&28802\end {array}\right ]$	$\left [\begin {array}{ll}2160&21 \\ 15&28856\end {array}\right ]$	$\left [\begin {array}{ll}937&5 \\ 8&32588\end {array}\right ]$	$\left [\begin {array}{ll}1281&6 \\ 10&32240\end {array}\right ]$
Reference used	mm10-3.0.0	mm10-3.0.0	GRCh38-3.0.0	GRCh38-3.0.0

We additionally show agreement on gene level (*gene-agreement*) in Table 5. For this we calculate for each method which genes are assigned to which cells (identified by their UMI). When creating the confusion matrix of two methods we identify all genes found by both methods. As with the cell-agreement matrices we filter any disagreements, where the expressed hit count does not exceed 1.0. Similar to the cell-agreement the gene-agreement is calculated as the count of cells found to contain a gene by both methods divided by the count of cells where at least one method found the gene present within a cell. Furthermore, in Table 6 the mean and standard deviation of the agreement matrices at cell-level and gene-level are listed for the different methods compared to RainDrop.

**Table 5:** Gene-agreement matrix (*G*) of each method compared to RainDrop for different datasets. Datasets are ordered by genome and size. Columns of the matrices show the average value of cells containing (left) or not containing (right) a gene calculated by RainDrop. Rows show the average count of cells containig (top) or not containing (bottom) a gene calculated by CellRanger (Alevin)

Method	$G_{neurons\_900}$	$G_{neuron\_9k}$	$G_{t\_4k}$	*G*_*pbmc*8*k*_
Alevin	$\left [\begin {array}{ll}179&8 \\ 1&1951\end {array}\right ]$	$\left [\begin {array}{ll}1136&22 \\ 9&10414\end {array}\right ]$	$\left [\begin {array}{ll}237&11 \\ 1&5207\end {array}\right ]$	$\left [\begin {array}{ll}469&38 \\ 2&9361\end {array}\right ]$
CellRanger	$\left [\begin {array}{ll}180&3 \\ 1&1954\end {array}\right ]$	$\left [\begin {array}{ll}1158&14 \\ 10&10398\end {array}\right ]$	$\left [\begin {array}{ll}236&3 \\ 2&5216\end {array}\right ]$	$\left [\begin {array}{ll}466&18 \\ 3&9383\end {array}\right ]$
Reference used	mm10-3.0.0	mm10-3.0.0	GRCh38-3.0.0	GRCh38-3.0.0

**Table 6:** Mean ± standard deviation of Cell-agreement (rows 1 and 2) and Gene-agreement (rows 3 and 4) of each method compared to RainDrop for different datasets. Datasets are ordered by genome and size

Method	$C_{neurons\_900}$	$C_{neuron\_9k}$	$C_{t\_4k}$	*C*_*pbmc*8*k*_
Alevin	0.97±0.010	0.97±0.010	0.96±0.013	0.95±0.012
CellRanger	0.98±0.006	0.98±0.005	0.99±0.005	0.99±0.003
Method	$G_{neurons\_900}$	$G_{neuron\_9k}$	$G_{t\_4k}$	*G*_*pbmc*8*k*_
Alevin	0.95±0.19	0.95±0.19	0.93±0.22	0.92±0.23
CellRanger	0.96±0.14	0.95±0.17	0.95±0.18	0.94±0.19
Reference used	mm10-3.0.0	mm10-3.0.0	GRCh38-3.0.0	GRCh38-3.0.0

To further emphasize that identified gene counts are biologically meaningful we have performed another test where we aggregated the genes of all cells and compared this distribution to an abundance estimation of bulk data from the same category (Accession numbers SRR1303990, SRR1373442, SRR1644186, SRR5074291 [17–20] for human and SRR327047, SRR3532922, SRR6753775 [21–23] for mouse). To process the bulk data we used RSEM (rsem-calculate-expression) which applies Bowtie2 for read mapping. To compare the single-cell data with the bulk mapping results we adopted the strategy proposed by Srivastava et al. [9]. For each single-cell result we aggregated the individual count of each individual gene over all cells to obtain the total gene count observed from the data. To quantify this count we calculated the Spearman correlation of the aggregated gene count distribution with the gene count distribution obtained from RSEM (Bowtie2). We excluded all genes that were found neither in the bulk nor in the single-cell data as this would unnecessarily increase correlation level for datasets with only a few genes present, overshadowing differences in the correlation of the identified data. The resulting correlation coefficients are shown in Table 7. We can see that a higher amount of data of the same species increases correlation levels. Overall correlations are very similar for the three tested tools, whereby RainDrop is highest on average.

**Table 7:** Spearman correlation against bulk datasets

Method	#Reads	RainDrop	Alevin	Cell Ranger
neurons_900	52.8M	0.793	0.790	0.790
neuron_9k	383.4M	0.834	0.831	0.820
t_4k	335.2M	0.741	0.722	0.740
pbmc_8k	784.1M	0.815	0.799	0.811

### Threshold evaluation

**Fig. 4:**
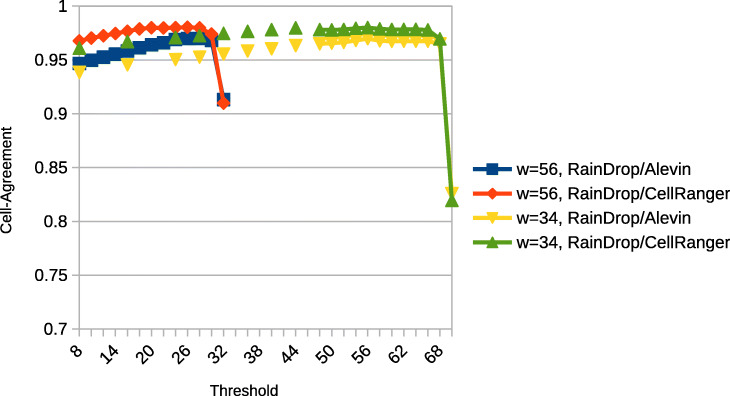
Mean Cell-Agreement for different thresholds and window sizes

**Fig. 5:**
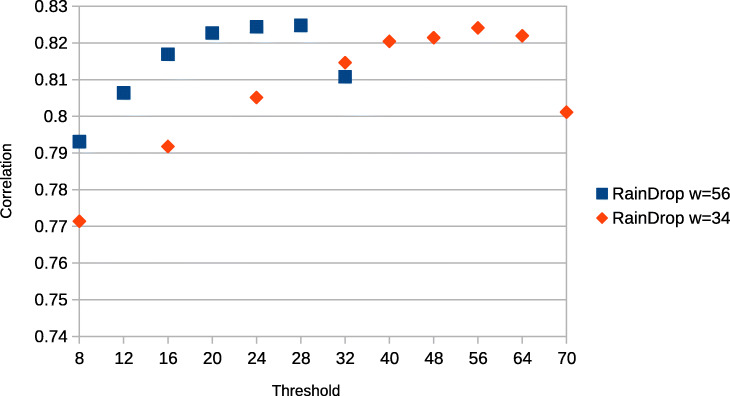
Spearman Correlation of RainDrop (dataset: neurons_900) against bulk-dataset (SRR3532922) for different thresholds and window sizes

We additionally varied the values for the threshold parameter and the window size used by RainDrop to evaluate their influence on the results. We used the neurons_900 dataset and tested different thresholds for window sizes of 56 and 34 with a constant sketch size of 16. The window size is applied to the reference as well as the read sequence to ensure a consistent sketching. In the first case the maximum amount of *k*-mers extracted as features in a read is 33 while in the second case this number increases to 70. Note that we therefore need different thresholds to accommodate for the different count of possible hits. To measure the results we used the average cell-agreement value (Fig. 4) and Spearman correlation with bulk data (Fig. 5). The results show that a low threshold only slightly reduces the agreement while the Spearman correlation is more affected. However, the overall gradient indicates that underestimation of the threshold near the peak is tolerable as the gradient here is not steep. However, choosing a threshold that is too high results in a drastic and more sudden drop of both agreement and correlation. In our tests this is only happening when the *k*-mer threshold is set very close to the maximum (32 or 70 respectively). Our results indicate that a threshold of approximately 80*%* of the maximal number of *k*-mers is a good choice.

## Discussion

Recent advances in single-cell transcriptomic sequencing have resulted in the production of large-scale NGS datasets that allow for the investigation of a number of biological questions. Parallelization of existing methods on compute clusters [24, 25] or GPUs [26] has been proposed to reduce corresponding runtimes. However, by applying efficient algorithmic techniques – like it is done in RainDrop – it is possible to perform important computational tasks such as the cell-to-gene count matrix generation on standard workstations without the need for specialized hardware platforms.

RainDrop is able to perform this task in substantially shorter time than its competitors by relying on exact substring matching with hash tables, subsampling of *k*-mers, and CPU multi-threading. Our performance evaluation further reveals that RainDrop achieves comparable mapping quality to both Cell Ranger and Alevin. Further attractive features of our implementation include thread scalability, low index memory consumption, and fast database creation times which allow for efficient execution on modern laptops and systems with large core counts.

Corresponding runtimes and memory requirements can be even further reduced by using smaller sketch sizes since this reduces both database size and lookups. However, this usually leads to a slightly lower sensitivity. The default parameters of RainDrop offer a good trade-off.

Choosing a low threshold to decide whether or not a read has enough hits to be considered to originate from a transcript can lead to a notable drop in accuracy as ambiguity increases which in return results in discarding lots of reads. Furthermore, choosing a very high threshold can also lead to a drastic decrease in accuracy as too many reads are discarded. Our results indicate that the latter is only a problem, when choosing a threshold that enforces a near 100*%* match of features. Thus, we have chosen a moderately high threshold. In general we propose a threshold of around 80*%* of the maximum *k*-mer count of a read. A larger window size decreases runtime as fewer features per read are detected at the cost of being a worse descriptor of the read leading to a lower accuracy. As a trade-off following the results of this paper we chose a window size of 56. This ensures that a read is only discarded if all candidate genes have a high probability of being the read’s origin, i.e., all candidate genes have a region that shares a very similar sketch with regions of another candidate gene. However, discarding these reads may in fact not drastically decrease the abundance estimation accuracy if enough reads are present since a truly expressed gene may be covered by other reads originating from different, less ambiguous regions of the gene.

Our results show that simply discarding ambiguous reads does not lead to notably lower mapping quality. We observed that RainDrop expresses similar or even slightly higher Spearman correlation compared to the bulk datasets than both Cell Ranger and Alevin. Furthermore, agreement matrices between different tools indicate high similarities for reads with a hit count greater than 1. Some of the differences can be explained by the utilized methods for whitelist generation.

## Conclusion

We have presented RainDrop – a method for fast gene-cell activation matrix computation from dscRNA-seq data together with a corresponding publicly available implementation. By relying on a big data approach based on *k*-mer subsampling we can scale efficiently towards large collections of single-cell transcriptomic sequencing data on standard workstations.

## Availability and requirements

Project name: RainDrop Project home page: https://gitlab.rlp.net/stnieble/raindropOperating system(s): Linux Programming language: C++ Other requirements: gccLicense: GPL-3Any restrictions to use by non-academics: according to license

## Data Availability

RainDrop is available at https://gitlab.rlp.net/stnieble/raindrop. The utilized Illumina single cell sequence read datasets can be downloaded from 10xGenomics at https://support.10xgenomics.com/single-cell-gene-expression/datasets. The used bulk datasets are available in the NCBI repository at https://www.ncbi.nlm.nih.gov/sra/SRR1303990, https://www.ncbi.nlm.nih.gov/sra/SRR1373442, https://www.ncbi.nlm.nih.gov/sra/SRR1644186, https://www.ncbi.nlm.nih.gov/sra/SRR5074291, https://www.ncbi.nlm.nih.gov/sra/SRR327047, https://www.ncbi.nlm.nih.gov/sra/SRR3532922, https://www.ncbi.nlm.nih.gov/sra/SRR6753775.

## Abbreviations

CB: Cellular bar code
UMI: Unique molecular identifier
dscRNA-seq - Droplet-based single-cell RNA-seq; COO: Coordinate format
